# The complete mitochondrial genome of *Pseudocolochirus violaceus* (Cucumariidae, Dendrochirotida)

**DOI:** 10.1080/23802359.2020.1788455

**Published:** 2020-07-14

**Authors:** Yan Wang, Ling Zeng, Jing Wen, Xuyan Li, Yafen Huang, Yulin Sun, Juan Zhao

**Affiliations:** a Department of Scientific Research, Lingnan Normal University, Zhanjiang, China; b Department of Chemistry, Lingnan Normal University, Zhanjiang, China; c Department of Biology, Lingnan Normal University, Zhanjiang, China

**Keywords:** *Pseudocolochirus violaceus*, mitochondrial genome, phylogeny

## Abstract

The complete mitochondrial genome of *Pseudocolochirus violaceus* was obtained and described in this study. This complete mitochondrial genome is 15,752 bp in length and consists of 13 protein-coding genes, 2 ribosomal RNA genes, and 22 transfer RNA genes. Except ND6 and 5 tRNAs, the others were encoded by the heavy strand. The overall base composition of the heavy-strand was 38.65% A, 12.09% G, 24.31% C, and 24.95% T, with a high A + T content of 63.6%. The phylogenetic analysis suggested that *P. violaceus* was closest to *Cucumaria miniata*. The newly described mitochondrial genome may provide valuable data for phylogenetic analysis for Holothuroidea.

Holothuroidea, as known as sea cucumber, is one of five echinoderms. It includes more than 900 described species in China (Liao 1997). As seafood, sea cucumbers are usually processed into a dried product known as ‘beche-de-mer’. They are regarded as traditional medicine, delicacies, and aphrodisiacs in Asia (Jaquemet and Conand 1999). *Pseudocolochirus violaceus*, also named sea apple cucumber, is a member of cucumaria. It is naturally distributed in the Indo-Pacific region, especially in Beibu Gulf of China, Sri Lanka, Vietnam, the red sea and Australia, and so on (Liao 1997). *Pseudocolochirus violaceus* was found mostly in sand-mud layer from 18 to 70 m deep in sea (up to 100 m) (Liao 1997). Dolmatov et al. (2012) found that *P. violaceus* was able to regenerate the anterior part of the body. Artificial breeding and larval rearing of *P. violaceus* was reported (Ajith Kumara et al. 2016). Unfortunately, only two DNA sequences (16 and 12 s) of *P. violaceus* could be found in GenBank. Lack of genetic resources has hindered conservation and utilization of *P. violaceus.* In this study, the complete mitochondrial genome of *P. violaceus* and its phylogenetic relationships within other sea cucumbers were investigated.

The specimen was collected from Shenzhen, Guangdong province, China (N22°35′, E114°31′) and kept in the Zoological Herbarium, Lingnan Normal University(Acc. Number SC20200319-3). The dorsal body muscles of *P. violaceus* were fixed in 100% ethanol and stored at −20 °C. Approximately 30 mg of muscle tissue was used for mitochondrial DNA(mtDNA) extraction with TIANamp Marine Animals DNA Kit (Tiangen, Beijing, China) according to the manufacturer’s specification. MtDNA was sequenced using the Illumina Hiseq Sequencing System (Illumina, San Diego, CA, USA). The clean data were acquired and assembled by the SPAdes and PRICE (Bankevich et al. 2012). BLAST (http://www.ncbi.nlm.nih.gov/BLAST/) and ORFs finder (https://www.ncbi.nlm.nih.gov/orffinder/) were used to identify and annotated protein-coding genes (PCGs). tRNAscan-SE 2.0 (Lowe and Chan 2016) was used to identified tRNA genes. A phylogenetic tree was constructed using MEGA 6.0 software.

The mitochondrial genome of *P. violaceus* is 15,752 bp in length (GenBank accession number: MT587564) and containing the typical set of 13 PCGs, 22 tRNA and 2 rRNA genes, and one putative control regions. The heavy strand consists of 38.65% A, 12.09% G, 24.31% C and 24.95% T bases, with a high A + T content of 63.60%. Of the 37 genes, 6 were encoded by the light strand, and 31 were encoded by the heavy strand. Eleven PCGs were initiated by ATG, and ND2 was started with GTG. Nine PCGs were terminate with the TAA as stop codon, while cox1, ND4 and ND6 were terminate with the TAG. Twenty-two tRNA genes, ranged in size from 65 to 73 bp.

The phylogenetic tree was constructed based on 13 concatenated PCGs from 11 sea cucumbers from GenBank database, by maximum likelihood (ML) method. *Salmacis bicolor rarispina* was used as an outgroup for tree rooting (Figure 1). It was demonstrated that *P. violaceus* was clustered with *Cucumaria miniata* firstly and both of them were clustered with *Colochirus quadrangularis*. This result suggested that the *P.violaceus* is closely related to *C. miniata* and *C. quadrangularis*. This newly reported genome of *P. violaceus* will contribute to future phylogenetic studies and population genetic analyses for *P. violaceus.*

**Figure 1.**
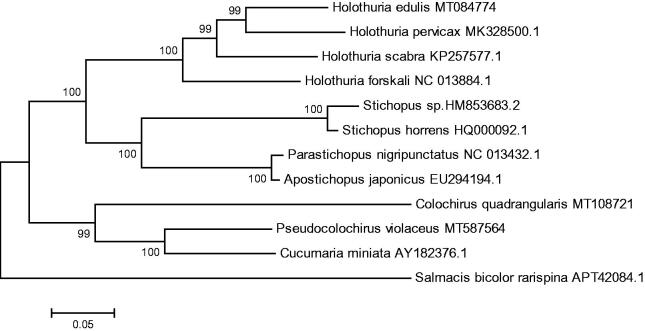
Phylogenetic tree of *Pseudocolochirus violaceus* and related species based on maximum likelihood (ML) method.

## Data Availability

The data that support the findings of this study are openly available in NCBI at https://www.ncbi.nlm.nih.gov/, reference number MT587564.
